# Serum Magnesium Levels and QTc Interval Prolongation As Prognostic Markers in Acute Myocardial Infarction: A Randomized Controlled Study

**DOI:** 10.7759/cureus.66051

**Published:** 2024-08-03

**Authors:** Venkat Naveen, Raji Rajesh Lenin, Lanord M Stanley, J S Kumar

**Affiliations:** 1 General Medicine, Sri Ramaswamy Memorial (SRM) Medical College Hospital and Research Centre, Chengalpattu, IND; 2 Medical Research, Sri Ramaswamy Memorial (SRM) Medical College Hospital and Research Centre, Chengalpattu, IND

**Keywords:** grace scoring, qtc prolongation, arrhythmias, magnesium, acute myocardial infarction

## Abstract

Introduction

Acute myocardial infarction (AMI) is frequently preceded by arrhythmias, which continue to be a prominent cause of abrupt fatality in AMI. Abnormal magnesium levels have been linked to the emergence of arrhythmia because it enhances myocardial metabolism and cardiac output and prevents calcium buildup and myocardial cell death by lowering arrhythmias. The objectives of this study were to evaluate serum magnesium levels and QTc interval as prognostic indicators in AMI patients during the initial 48 hours of hospital stay and to correlate these parameters with the Global Registry of Acute Coronary Events (GRACE) scoring. We studied AMI patients by dividing them into two groups: those with abnormal and those with normal serum magnesium levels.

Methods

After obtaining ethical approvals, patients were subjected to detailed history, which included sociodemographic details, drug history, clinical examination, and investigations such as creatine kinase myocardial band (CK-MB), CK-total, troponin-T, ECG (QTc interval), two-dimensional echocardiogram (2D-ECHO), serum creatinine and magnesium levels, heart rate, and blood pressure. We also calculated the GRACE score for all patients.

Results

We found that patients in the age group of 51-60 years were more prone to developing arrhythmias, and while AMI was more prevalent in males, the occurrence of arrhythmias was slightly higher in females with AMI. Anterior wall motion abnormality (AWMA) was the most predominant abnormality, and 12.3% of AWMA patients had arrhythmias. QTc interval was significantly longer in patients who developed arrhythmias. Interestingly, among patients with QTc prolongation, 35% patients had abnormal magnesium levels, while 65% had normal magnesium levels. In our study, of the 25 patients with hypermagnesemia, nine (36%) developed arrhythmias, while of the 75 patients with hypomagnesemia, 15 (20%) patients developed arrhythmias. Interestingly, we found that there was a positive correlation between GRACE score and serum magnesium as well as QTc interval prolongation. Lastly, among the six deaths reported, three (50%) patients had arrhythmias.

Conclusion

Overall, we conclude that serum magnesium levels play a pivotal role as a prognostic tool for arrhythmias and are a useful investigation during the initial 48 hours of admission in AMI patients.

## Introduction

The second most prevalent intracellular ion is magnesium. It functions as a cofactor in more than 300 enzymatic processes, including those that affect lipid peroxidation, glycemic regulation, and blood pressure. As a result, the cardiovascular system also depends on it [1]. The average human body has 24g of magnesium, of which 50-60% is found in bones and the remaining 25-30% in soft tissues, and less than 1% of the body's magnesium is found in the serum [2].

Magnesium has been linked to the emergence of arrhythmias and other complications of acute myocardial infarction (AMI) as well as other cardiovascular disorders. Magnesium ions are thought to be necessary for preserving the myocardium's structural and functional integrity. It was observed that the myocardial magnesium concentration was extremely low in patients who died suddenly from ischemic heart disease (IHD) [3]. Ventricular fibrillation and coronary vasospasm, brought on by magnesium shortage, both result in sudden mortality in IHD. Magnesium deficiency has also been linked to the development of atheromatous plaques due to its association with hyperlipidemia [4]. Importantly, myocardial infarction (MI) is among the leading causes of death today, and its prognostication depends on several different variables, many of which are yet to be known [5].

Magnesium plays an important role in action potentials and electrical conductance. Phase 4 of the action potential is when potassium ions (K+) move inward through channels, and this process depends on magnesium, such that when magnesium is depleted, this inward flow is inhibited [3-5]. This results in inadequate function of the Na+/K+ pump, and the K+ depletion leads to intracellular Na+ aggregation. Moreover, the degree of cardiac ischemia and the effect of catecholamines are both impacted by magnesium regulation of Ca2+ entry into the mitochondria. Magnesium lessens the amount of calcium that enters the cells during ischemia and shields the myocardium from ischemia-related harm. Although the reduction of extracellular magnesium does not perturb the electrophysiology of myofibrils, in vitro models of early potentials have shown that adding magnesium blocks the automatic activity [6].

By altering the amounts of other ions that have an impact on heart mechanics, exogenous magnesium reduction primarily impacts cardiac excitability and contractility. Most laboratory studies in solitary ventricular tissue or myocytes find an inverse relationship between magnesium content and inotropic response, which is likely caused by increased Ca2+ in flow that encourages sarcoplasmic reticular Ca2+ release [6]. Once it got established that magnesium was beneficial for numerous arrhythmias, notably “tachy and brady arrhythmias,” it has been grossly utilized in general practice. Recently, a number of research studies have suggested that magnesium can be more effective for the treatment of some life-threatening arrhythmias [7]. It is uncertain if the persistent hypomagnesemia found in AMI patients is caused by the increased cardiovascular hazard or due to the generation of catecholamines that promotes lipolysis [8].

The time between the start of the QRS complex and the end of the T wave is known as the QT interval. It largely depicts the stimulated ventricles returning to the resting state and is inversely influenced by the heart rate. Longer QT intervals can be caused by certain electrolyte abnormalities, such as reduced magnesium, potassium, and calcium concentrations [9]. Overall, the aims of this study were to evaluate serum magnesium levels and QTc interval as prognostic indicators in AMI patients during the initial 48 hours of hospital stay and to correlate these parameters with the Global Registry of Acute Coronary Events (GRACE) scoring. We believe that early intervention to treat abnormal magnesium levels can improve electrical stability and halt the QTc interval prolongation, thereby reducing the risk of life-threatening arrhythmias and in-hospital mortality rates in AMI patients. In summary, magnesium would be strongly indicated in patients with QTc prolongation, high GRACE scores, both hypo- and hypermagnesemia, those post-AMI, those who have developed arrhythmias, and those at high risk of mortality due to arrhythmias. Regular monitoring and appropriate management of magnesium levels in these groups could help in reducing the incidence and severity of arrhythmias and potentially improve clinical outcomes.

## Materials and methods

Institutional Ethical Committee approval from SRM Medical College Hospital and Research Centre was obtained before initiating this study (ethical clearance number: 2391/IEC/2021). Acute myocardial infarction (AMI) patients aged 20 to 80 years presenting to the emergency department and cardiac ICU of SRM Medical College Hospital and Research Center from January 2021 to August 2022 with symptoms of ischemia, ECG changes, and raised cardiac biomarkers were considered as the study population. The American Heart Association (AHA) and the European Society of Cardiology guidelines [10,11] were followed for the diagnosis of AMI. A total of 200 AMI patients were selected for the study and were divided into two groups. The first group consisted of 100 patients who had abnormal serum magnesium levels (both hypomagnesemia and hypermagnesemia) and whose magnesium levels were corrected during hospital stay. The second group consisted of 100 patients who had normal serum magnesium levels. Patients with hypokalemia (K+), hypocalcemia (Ca2+), renal failure, chronic diarrhea, and liver cirrhosis, as well as patients on diuretics, long-term antacids, and anti-cancer drugs were excluded from the study.

The above groups were divided using the randomization method after checking serum magnesium levels during the time of admission and were followed up for the next 48 hours in hospital stay for QTc prolongation and arrhythmias and further correlating these parameters with the GRACE score for in-hospital mortality and risk stratification in both groups.

After obtaining consent from the patient and the patient’s attender, patients were subjected to detailed history, which included sociodemographic details, drug history, clinical examination, and investigations such as creatine kinase myocardial band (CK-MB), CK-total, troponin-T, ECG (QTc interval), two-dimensional echocardiogram (2D-ECHO), serum creatinine and magnesium levels, heart rate, and blood pressure.

We also calculated the GRACE score for all patients. The primary purpose of the GRACE score was to anticipate in-hospital death in ACS patients, as it provides the most accurate stratification for in-hospital deaths in AMI patients [12-14]. According to the scores, patients were classified into the following three categories of risks: low (<109), intermediate (109-140), and high (>140).

Data were entered into MS Excel (Microsoft Corp., Redmond, WA) and analyzed in SPSS Version 24 (IBM Corp., Armonk, NY). Descriptive statistics were represented as percentages, mean with SD, or median with IQR, depending on nature of the data. The Shapiro-Wilk test was applied to find normality. The chi-square test, independent t-test, Mann-Whitney U test, and Fisher exact test were calculated, and p<0.05 was taken as significant statistically.

## Results

Out of a total number of 200 participants, 30 (15%) patients had arrhythmias during the 48 hours of hospital stay, while 170 (85%) did not. We began our analysis by looking at various sociodemographic and medical parameters in our study group and understanding the association of these factors with arrhythmias. These data are presented in Table 1. Patients in the age group of 51-60 years were most prone to developing arrhythmias (33.3%). Even though we predominantly had male patients in our study (136 males), only 18 (13.2%) of them developed arrhythmias, whereas 18.8% (12 out of 64) females in our study developed arrhythmias. Interestingly, majority (43.3%) of the patients who developed arrhythmias had normal blood pressure, but around 50% of the patients who did not develop arrhythmias had high blood pressure. We also performed 2D-ECHO to identify the type of regional wall motion abnormalities in AMI patients. Based on the ECHO, we determined that anterior wall motion abnormality (AWMA) was the most predominant abnormality (n=114) in the study population. Among patients who had arrhythmias, 46.7%) had AWMA, followed by lateral wall motion abnormality (LWMA), which was seen in 33.3% of patients. The chi-square analysis revealed that there was no significant association between the occurrence of arrhythmias, and age, sex, blood pressure, or motion abnormalities (Table 1).

**Table 1:** Association of sociodemographic and medical parameters with arrhythmia in acute myocardial infarction patients. Qtc, corrected QT interval; ECHO, echocardiography; AWMA, anterior wall motion abnormality; IWMA, inferior wall motion abnormality; LWMA, lateral wall motion abnormality; ALWMA, anterolateral wall motion abnormality; ILWMA, inferolateral wall motion abnormality; GRACE, Global Registry of Acute Coronary Events

Parameters	Range	Yes (n=30)	No (n=170)	P-value
Age	33-40	0 (0%)	7 (4.1%)	0.541
	41-50	7 (23.3%)	29 (17.1%)	
	51-60	10 (33.3%)	44 (25.9%)	
	61-70	8 (26.7%)	63 (37.1%)	
	71-80	5 (16.7%)	27 (15.9%)	
Sex	Male	18 (60%)	118 (69.4%)	0.396
	Female	12 (40%)	52 (30.6%)	
Blood pressure	90-60	4 (13.3%)	17 (10%)	0.213
	91-120/61-80	13 (43.3%)	45 (26.5%)	
	121-130/81-90	3 (10%)	24 (14.1%)	
	>130/90	10 (33.4%)	84 (49.4%)	
QTc interval	Normal	1 (3.3%)	57 (33.5%)	0.0003
	Prolonged	29 (96.7%)	113 (66.5%)	
ECHO	AWMA	14 (46.7%)	100 (58.8%)	0.228
	IWMA	4 (13.3%)	28 (16.5%)	
	LWMA	10 (33.4%)	40 (23.5%)	
	ALWMA	1 (3.3%)	1 (0.6%)	
	ILWMA	1 (3.3%)	1 (0.6%)	
Mg2+	Hypo	15 (50%)	60 (35.3%)	0.0003
	Normal	6 (20%)	94 (55.3%)	
	Hyper	9 (30%)	16 (9.4%)	
GRACE	Low: <109	1 (3.3%)	6 (3.5%)	0.860
	Intermediate: 109-140	4 (13.3%)	17 (10%)	
	High: >140	25 (83.4%)	147 (86.5%)	
Deaths	Yes	3 (10%)	3 (1.8%)	0.045
	No	27 (90%)	167 (98.2%)	

**Figure 1:**
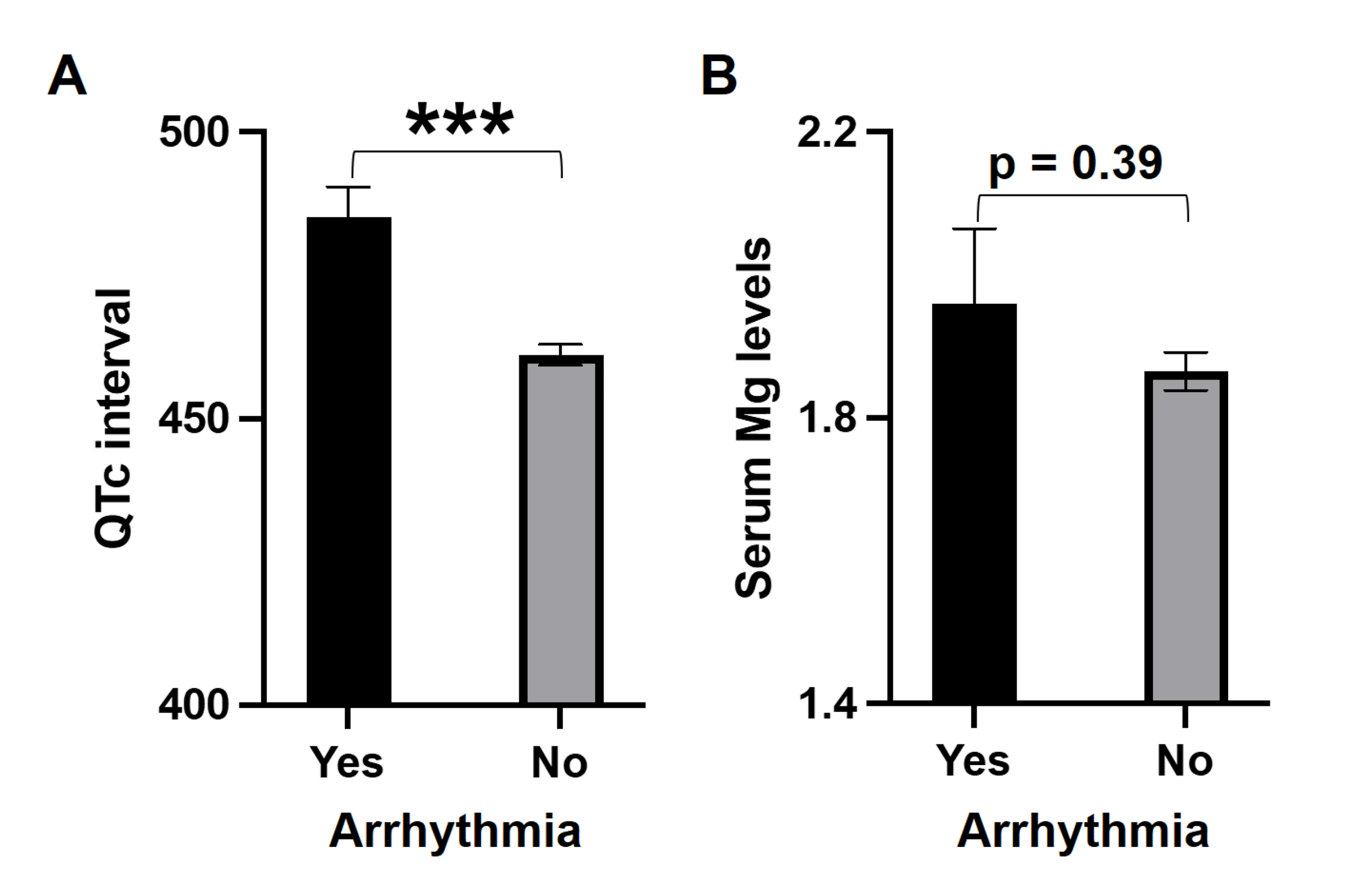
Comparison of (A) QTc interval and (B) serum magnesium levels in patients with or without arrhythmia.

Next, we looked at the QTc intervals and found that the mean QTc interval was significantly higher in patients who had arrhythmias than those without (485.1 ± 5.4 vs 461.1 ± 1.8, p=0.0001, Student’s t-test; Figure 1A). Furthermore, of the 30 patients who developed arrhythmia, 29 (96.7%) patients had QTc interval prolongation, demonstrating a statistically significant (p=0.0003) association between arrhythmias and QTc prolongation (Table 1). Similarly, we checked the magnesium levels in our study participants and found that there was no difference in the serum magnesium in patients with or without arrhythmias (1.96 ± 0.1 vs 1.86 ± 0.03, p=0.387, Student’s t-test; Figure 1B). However, the chi-square test revealed a significant association between the occurrence of arrhythmias and serum magnesium levels (p=0.003; Table 1). Among the study population, six deaths were reported, of which three patients had arrhythmias and three did not, and the association between arrhythmias and death was statistically significant (p = 0.045).

One of the major objectives of our study was to determine whether serum magnesium level can be used as a prognostic indicator in AMI patients. The normal range of serum magnesium was 1.7 to 2.4 mg/dL, and in our study, 100 patients had normal magnesium levels and 100 had abnormal levels, of which 25 patients had hypermagnesemia and 75 patients had hypomagnesemia. Surprisingly, of the patients with normal magnesium levels, 92% had QTc prolongation, but only 50% of the patients with abnormal magnesium levels had QTc prolongation (Table 2). In terms of GRACE score, 95% of patients with normal and 77% patients with abnormal serum magnesium levels had a high GRACE score (Table 2). Lastly, while 4% of patients with normal magnesium levels had an adverse outcome of death, only 2% patients with abnormal magnesium died (Table 2). Overall, there was a significant association of serum magnesium levels with both QTc intervals as well as GRACE score (chi-square test p<0.0001 and p=0.0007, respectively), but not with death (chi-square test p=0.682).

**Table 2:** Association of QTc interval, GRACE score, and death rate with magnesium levels in acute myocardial infarction patients. GRACE, Global Registry of Acute Coronary Events

Parameters	Range	Normal (n=100)	Abnormal (n=100)	P-value
QTc interval	Normal	8	50	<0.0001
	Prolonged	92	50	
GRACE	Low: <109	0	7	0.0007
	Intermediate: 109-140	5	16	
	High: >140	95	77	
Deaths	Yes	4	2	0.682
	No	96	98	

**Figure 2:**
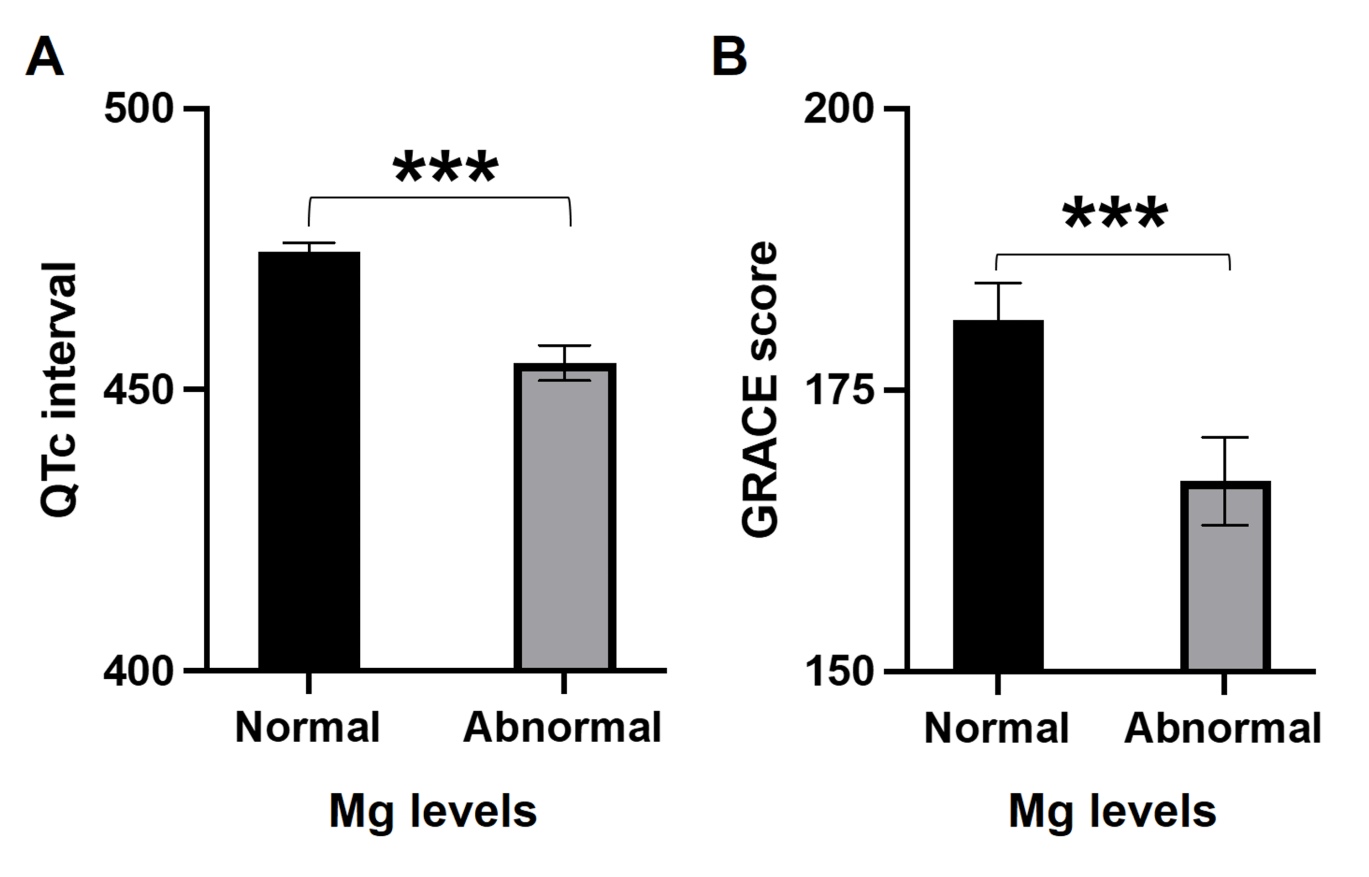
Comparison of (A) QTc interval and (B) GRACE score in patients with normal and abnormal serum magnesium levels. GRACE, Global Registry of Acute Coronary Events

We found very surprising results in terms of the values of QTc intervals and GRACE score in patients with normal versus abnormal magnesium levels. The mean QTc interval was significantly higher in patients who had normal magnesium levels compared to patients with hypermagnesemia or hypomagnesemia (474.6 ± 1.5 vs 454.7 ± 3.1, p<0.0001, Student’s t-test; Figure 2A). Similarly, we calculated the GRACE score in our study participants and found that patients with normal magnesium levels had a significantly higher GRACE score compared to patients with abnormal magnesium levels (181.2 ± 3.3 vs 166.9 ± 3.9, p=0.006, Student’s t-test; Figure 2B). 

**Figure 3:**
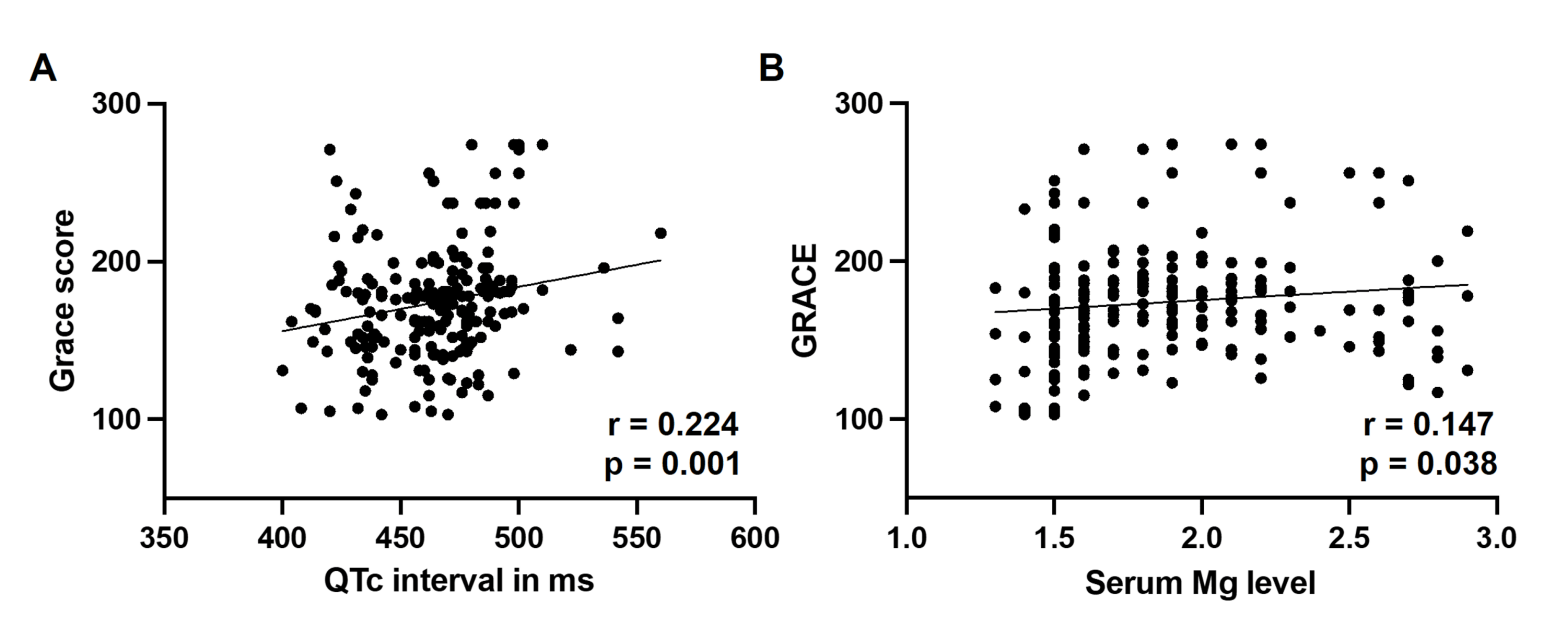
Comparison of (A) QTc interval and (B) GRACE score in patients with normal and abnormal serum magnesium levels. GRACE, Global Registry of Acute Coronary Events

Next, we wanted to determine the correlation of QTc interval values and serum magnesium levels with GRACE score, which provides an accurate stratification for in-hospital deaths in AMI patients. Analyses showed a significant correlation between these two parameters and severity of GRACE score. Specifically, scatter diagrams were plotted to visualize the correlation between GRACE score and QTc interval and magnesium levels (Figure 3). QTc interval (r=0.224, p=0.001; Figure 3A) and serum magnesium levels (r=0.147, p=0.038; Figure 3B) increased consistently as the risk for in-hospital mortality, indicated by GRACE score, became more severe. Overall, these results show that both QTc prolongation and magnesium levels correlate with AMI severity and suggest that this can be used as a prognostic marker in AMI patients. In summary, for chronic kidney disease patients with AMI, maintaining serum magnesium within the normal range (1.7 to 2.4 mg/dL) is crucial, with careful monitoring to avoid both hypo- and hypermagnesemia, while also considering the overall electrolyte balance and renal function.

## Discussion

The major goal of our study was to evaluate serum magnesium levels and QTc interval as prognostic indicators in AMI patients during the initial 48 hours of hospital stay and correlate these parameters with GRACE scoring. A total of 200 patients were included in the final analysis. The mean age of patients who developed arrhythmias was 59.7 ± 1.9,5 and patients in the age group of 51-60 years were most prone to developing arrhythmias. Among our study population, 64 female patients were included, of which 12 (18.8%) patients developed arrhythmias and 136 male patients were included, of which 18 (13.2%) developed arrhythmias. These results suggest that while males are more vulnerable to developing myocardial infarcts, but females have a slightly higher risk for developing arrhythmias secondary to AMI. We also found that the most common wall motion abnormality in our study population was AWMA, which was seen in 114 patients, of which 14 (12.3%) patients developed arrhythmias, followed by LWMA, which was seen in 50 patients, of which 10 (20%) patients had arrhythmias. With the above results, we concluded that AWMA is more profound in this study.

The first parameter of interest in our study was the QTc interval. In patients who developed arrhythmia, the mean QTc interval was found to be 485.1 ± 5.35, with the minimal interval being 429 ms and the maximum being 560 ms. In this study, a total of 142 patients had QTc interval prolongation, of which 29 (20.4%) patients developed arrhythmias, and we found that there was a statistically significant association between QTc interval and arrhythmias (p<0.001). Moreover, in terms of magnesium levels, 50% of the patients with abnormal magnesium levels and 92% of the patients with normal magnesium levels had QTc interval prolongation, and there was also a statistically significant association between these two parameters (p<0.001). In patients who developed arrhythmias, the mean value for GRACE score was 176.2 ± 7.12, with the minimum score being 105 and the maximum score being 274. Straus et al. studied 125 individuals who had an average follow-up of 6.7 years and reported that abrupt cardiac death was the primary outcome and that a threefold greater risk of sudden cardiac death was linked to an unusually extended QTc interval (>460 ms in women and >450 ms in men) (hazard ratio, 2.50; 95% CI, 1.3 to 4.7) [15].

The second parameter of interest in our study was the serum magnesium levels, and in patients who developed arrhythmias, the mean serum magnesium level was 1.96 ± 0.1, with the minimum value being 1.3 mg/dL and the maximum being 2.9 mg/dL. Of the 25 patients with hypermagnesemia, nine (36%) patients developed arrhythmias, and of the 75 patients with hypomagnesemia, 15 (20%) patients developed arrhythmias. The association between arrhythmias and magnesium level was found to be statistically significant (p=0.0003). Similar results were observed in another study by Rasmussen et al., in which 273 people with probable AMI were randomly assigned to receive IV magnesium or a placebo [16]. Comparing the magnesium group to the placebo group, they found a statistically significant reduction in ventricular arrhythmia (p<0.05). Our correlation analysis found a positive link between GRACE score and serum magnesium levels (p<0.038) levels as well as with QTc interval prolongation (p<0.001).

Looking at the association between GRACE score and serum magnesium levels, we found that 95% of the patients with normal magnesium and 77% of patients with abnormal magnesium levels had a high-risk GRACE scoring, while 5% patients with normal magnesium levels and 16% patients with abnormal magnesium levels had a GRACE score that was in the intermediate risk category. Despite this significant association, there was no significant correlation between GRACE score and arrhythmias (p=0.86), but higher GRACE scores can lead to increased in-hospital mortality in AMI subjects. Another study by Tscherny et al. found that GRACE risk scores were superior to CRUSADE (Can Rapid risk stratification of Unstable angina patients Suppress ADverse outcomes with Early implementation of the ACC/AHA guidelines) scores (receiver-operator distinctive “area under the curve” 0.91 for GRACE score compared to 0.83 for CRUSADE; p<0.001) in predicting in-hospital mortality among AMI patients [17].

Finally, considering the adverse outcome of death, we noted that among our study population of 200, six deaths were reported, of which three (50%) patients had arrhythmias, establishing a statistically significant association between arrhythmias and death (p=0.045). However, with respect to magnesium levels, four (4%) patients with normal magnesium levels and two (2%) patients with abnormal magnesium levels died, suggesting that there is no correlation between magnesium levels and death (p=0.68). It is important to emphasize that among the patients with abnormal magnesium levels, who got medically intervened by correcting serum magnesium, there were two deaths, whereas there were four deaths in patients without magnesium intervention.

Our study does have some limitations that need to be acknowledged. The sample size in our study was only 200, which is small, and thus our results cannot be extrapolated to the general population. It was a single-center study, and thus larger multicenter studies are required to reach appropriate conclusions. Lastly, our follow-up duration was restricted to 48 hours, which is a short time, and longer follow-up durations are required to get a complete understanding of the correlation of arrhythmias and adverse outcomes with QTc intervals and serum magnesium levels.

## Conclusions

With the above evidence, we can draw conclusions that early intervention of abnormal serum magnesium levels can improve electrical stability and halt QTc interval prolongation, thereby reducing the risk of life-threatening arrhythmias and in-hospital mortality among AMI patients. However, it is important to remember that abnormal serum magnesium levels are not the only causative factors for arrhythmias, and future studies must look at other structural abnormalities. Hence, serum magnesium levels play a pivotal role as a prognostic tool for arrhythmias and, a useful investigation during the initial 48 hours of admission in AMI patients so that magnesium level corrections can be considered.
